# A comprehensive dataset on political strikes in Latin America: Event characteristics, mobilization dynamics, and socio-political contexts (1990–2020)

**DOI:** 10.1016/j.dib.2025.112180

**Published:** 2025-10-15

**Authors:** Rodrigo M. Medel

**Affiliations:** Facultad de Gobierno, Universidad de Chile, Santiago, Chile

**Keywords:** Labor protests, Political strike, Collective action, Social movements, Strike dynamics, Latin American politics, Labor conflicts

## Abstract

Political strikes—defined as collective work stoppages by workers opposing or challenging government policies—offer valuable insights into the dynamics of social and political conflict. These events lie at the intersection of labor mobilization, state-society relations, and governance challenges. The dataset *Political Strikes in Latin America (1990–2020)* was developed to enhance the study of these phenomena across 18 Latin American countries over three decades. It systematically categorizes strikes into three types: general political strikes involving workers from multiple economic sectors, sectoral political strikes focused within specific sectors, and local political strikes limited to single companies.

The dataset was constructed using >3000 press reports on political strikes sourced from the LatinNews media outlet. The data underwent a rigorous validation through intercoder reliability checks and cross-referencing with external sources to ensure reliability and accuracy. This process identified and coded 195 general political strikes, 507 sectoral political strikes, and 263 local political strikes. The dataset comprises 73 variables capturing the internal mobilization dynamics of strikes—including temporality, geography, social organization, communication, disruption, tactics and targets, repression, and negotiation—and the broader political and economic contexts in which these events occurred.

Its comprehensive structure and rich array of variables make it well-suited for analyzing labor movements, political crises, the dynamics of collective action in Latin America and their implications for governance and public policy.

Specifications Table

**Table:** 

Subject	Social Sciences
Specific subject area	Political Science / Sociology
Type of data	*Excel file, CSV file, and codebook (PDF).*
Data collection	The dataset was constructed using press reports from the *LatinNews* media outlet, including its *Latin American Weekly Report* and *LatinNews Daily* bulletins. The data collection process combined automated searches for key terms such as ``strike,'' ``protest,'' and ``stoppage'' with meticulous manual reviews to address potential gaps in coverage. A team of researchers identified the news items corresponding to each unit of analysis: political strikes. Using a codebook developed through both deductive and inductive approaches, the team systematically coded the material, applying clear rules to assign numerical values to various pieces of information about political strikes. Validation procedures ensured the accuracy and consistency of the dataset through intercoder reliability checks and cross-referencing with external databases. The data were further standardized by organizing variables into a structured framework of 73 categories, as defined in the comprehensive codebook. This systematic approach ensured consistency and reliability in the organization and interpretation of the data.
Data source location	The dataset is stored at Universidad de Chile, Faculty of Government, Santiago, Chile
Data accessibility	The dataset is uploaded on Harvard Dataverse Repository name: Political Strikes in Latin America (1990–2020) Data identification number: https://doi.org/10.7910/DVN/YZRT6V Direct URL to data: https://doi.org/10.7910/DVN/YZRT6V

## 1 Value of the Data

• These data provide a comprehensive, longitudinal view of political strikes in Latin America, covering 18 countries over 31 years (1990–2020). The detailed coding of 73 variables allows for an in-depth analysis of strike characteristics, such as temporality, geography, tactics, and repression, making it a robust resource for examining labor mobilization and state-society dynamics in the region.
• The dataset stands out because it focuses on general, sectoral, and local political strikes, a less-explored area in labor studies. It includes validated information cross-referenced with external datasets, ensuring accuracy and consistency, and it applies a standardized methodology that can be replicated in other regions.
• Scholars in political science, sociology, labor studies, and Latin American studies can utilize these data to investigate topics such as political mobilization, state responses to protests, and the role of unions in political contention. It also holds relevance for policymakers analyzing labor conflicts and social stability. The data can also be integrated into cross-national studies on protest movements, governance, and political crises.
• The dataset provides a foundation for examining the broader implications of labor mobilization, such as its influence on policy change and democratization processes. Its detailed structure also enables the creation of derived variables and indices for quantitative analyses in related fields.
• To facilitate compatibility with the country-year structure of datasets such as V-Dem and WhoGov, we provide a ready-to-use R function (link_political_strikes_to_vdem_whogov()) that aggregates the event-level political strike data into a country-year panel and assigns standardized ISO codes. This function is publicly available in the Dataverse repository of the Political Strikes Database, alongside a technical appendix that offers a step-by-step guide for its correct implementation and use.

## 2 Background

The dataset ``Political Strikes in Latin America (1990–2020)'' [1] was developed as part of a broader research effort to address the gap in systematic quantitative studies on political strikes in Latin America. Its conceptual framework draws on theoretical distinctions between economic and political strikes [2]. Economic strikes generally target public or private sector employers, with demands centered on working conditions or employment terms. Political strikes, by contrast, aim to contest government policies, often reflecting broader conflicts between unions and governments over public issues [3].

While labor strikes, encompassing both economic and political dimensions, have long been a subject of scholarly attention in the social sciences [4], quantitative research in Latin America has predominantly focused on economic strikes. This bias stems from official records in the region, which exclusively document economic strikes (usually at the company level) while neglecting political strikes. By comparison, Europe has made significant strides in the quantitative study of political strikes [5].

This oversight in Latin American research is particularly significant given the historical role of political strikes in shaping public policy, resisting neoliberal reforms, and influencing democratization processes. By providing systematic data on political strikes, this dataset contributes to understanding their broader impact and addresses a notable omission in the study of labor mobilization in the region.

## 3 Data Description

The dataset on political strikes in Latin America encompasses data from 18 countries between 1990 and 2020. It includes 965 observations, with each entry representing a political strike in a specific country at a given time, and contains 73 variables. Political strikes are classified into three levels based on their scope: local, sectoral, and general [3].

1. **Local Political Strikes:** These strikes target political power at a localized level, often involving one or a small group of companies (usually large or strategically significant). For instance, workers from an innovative company may mobilize against state-imposed labor regulations.
2. **Sectoral Political Strikes:** These strikes occur within a single economic sector, often in response to government policies affecting that sector. A typical example would be strikes led by a mining sector federation pressing for legislative reforms on issues impacting the industry.
3. **General Political Strikes:** These involve the simultaneous stoppage of multiple economic sectors (two or more), often gaining national significance. Such strikes typically challenge public policies or government control and are led by macro-union organizations, such as federations or confederations of workers.

Table 1 provides descriptive statistics for these three strike types. It includes the mean and standard deviation of strike duration (in days), the mean and standard deviation of the number of news reports covering the strikes, and the total number of events for each type. Sectoral strikes are the most frequent (507), followed by local strikes (263) and general strikes (195). This detailed classification enhances the dataset's utility for understanding the diverse forms and dynamics of political strikes across Latin America.

**Table 1:** Descriptive statistics by type of political strike.

Type	Duration in days (Mean)	Duration in days (SD)	Number of news reports (Mean)	Number of news reports (SD)	N
Local Strike	10.26	14.23	1.72	1.42	263
Sectoral Strike	14.98	22.21	2.37	2.23	507
General Strike	9.83	23.07	3.84	4.61	195

Means, Standard Deviations, and Sizes.

**Fig. 1:**
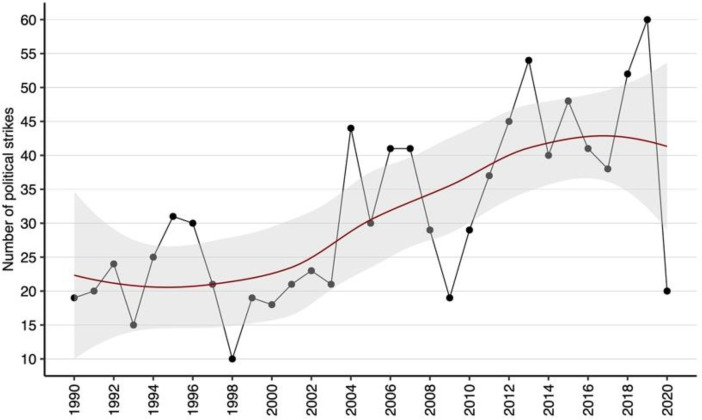
Temporal trend of political strikes (1990–2020).

Fig. 1 illustrates the temporal trend in the annual number of political strikes across Latin America from 1990 to 2020. The chart includes a fitted trendline with a shaded confidence interval, providing a visual representation of changes in strike activity over time. Peaks in certain years highlight fluctuations in political contention, reflecting periods of heightened mobilization.

**Fig. 2:**
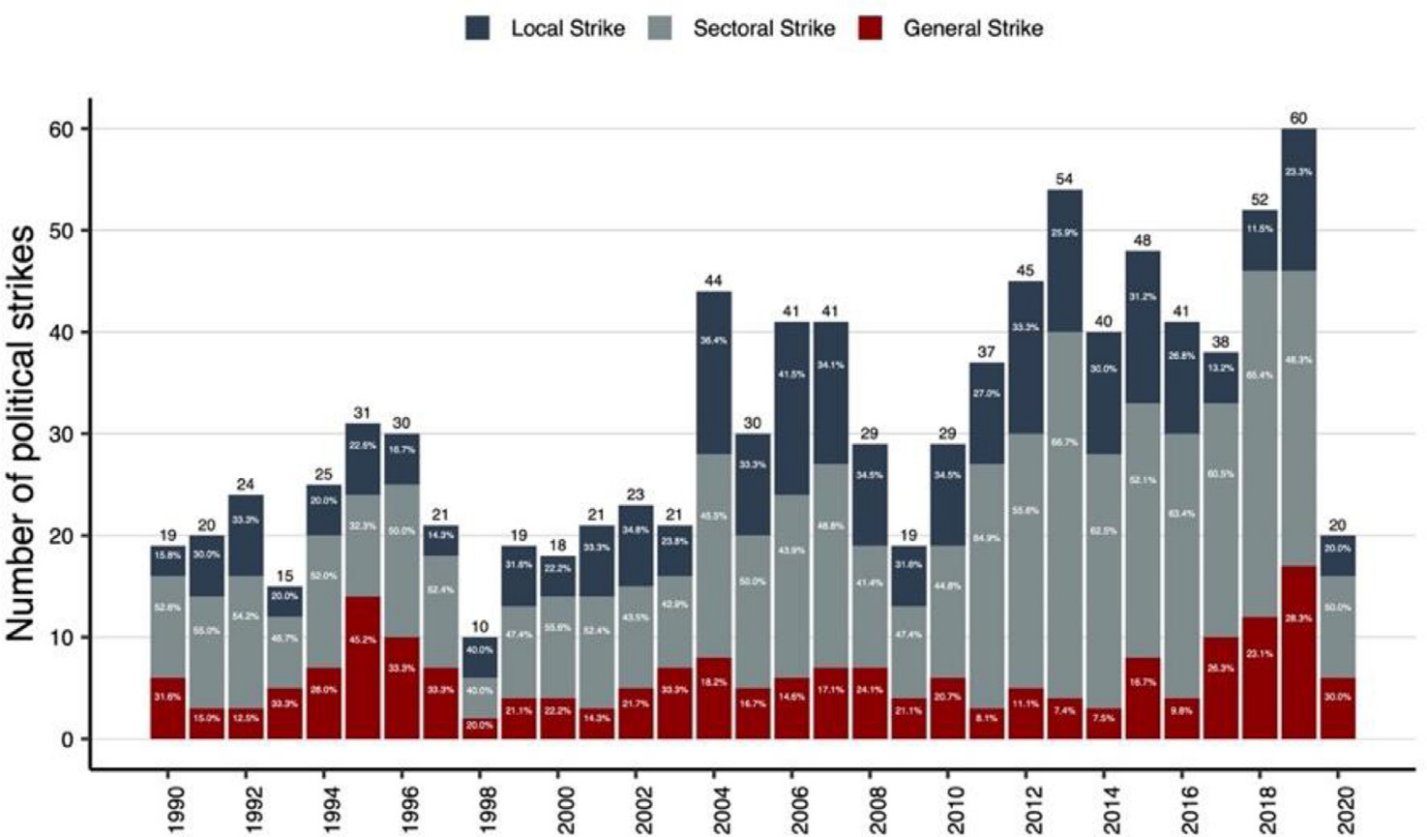
Political strikes by year and type (1990–2020).

Fig. 2 presents a stacked bar chart displaying the annual number of political strikes in Latin America from 1990 to 2020, categorized by type: local, sectoral, and general strikes. This visualization highlights variations in strike activity over time, revealing the predominance of sectoral strikes in most years. The chart offers a clear overview of temporal changes in the intensity and composition of labor mobilization during the study period.

**Fig. 3:**
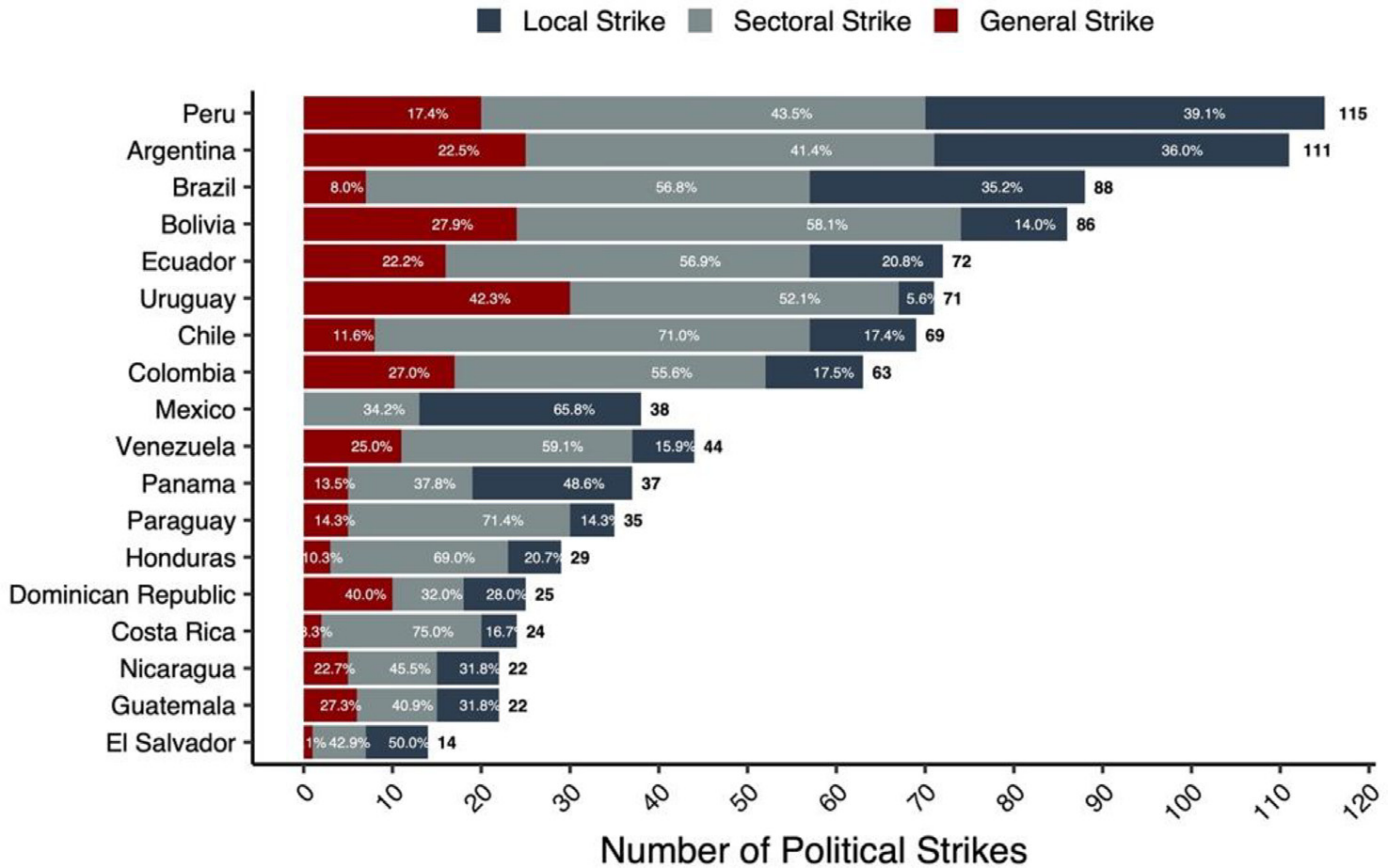
Distribution of political strikes by country and type (1990–2020).

Fig. 3 provides a horizontal bar chart illustrating the distribution and frequency of political strike types across 18 Latin American countries from 1990 to 2020. Each bar displays the total number of strikes and the proportional breakdown by type—local, sectoral, and general. The chart highlights significant variation in strike activity among countries, with Peru, Argentina, and Brazil registering the highest total numbers of strikes. The distribution of strike types also varies widely, reflecting differences in labor mobilization strategies and political contexts across the region.

Table 2 provides a detailed overview of the variables related to social organization, emphasizing the involvement of various organizations and support structures in political strikes. The data include the presence of neighborhood organizations, student organizations, and international organizations. Notably, political parties supported 12.5 % of the strikes, while the country’s primary union organization was involved in 27.7 % of cases.

**Table 2:** Social organization variables.

Variable	Description	Category	Frequency	Proportion
actsoc1	Presence of neighborhood organizations	1 = Yes	114	12.6 %
		2 = No	789	87.4 %
actsoc2	Presence of student organizations	1 = Yes	137	15.2 %
		2 = No	766	84.8 %
actsoc3	Presence of international organizations	1 = Yes	20	2.2 %
		2 = No	883	97.8 %
apoyopartidos	Political parties support the call for a general strike	1 = Yes	113	12.5 %
		2 = No	790	87.5 %
central	Support from the country's top union organization	1 = Yes	250	27.7 %
		2 = No	653	72.3 %
centralesotras	Support from other top-level union organizations (central or other denomination)	1 = Yes	419	46.4 %
		2 = No	484	53.6 %
tiporg	Maximum union organization in the call	Temporary macro-organization	57	6.3 %
		Socio-union movement(s)	179	19.8 %
		Trade Union Center	148	16.4 %
		Confederation(s)	128	14.2 %
		Federation(s)	89	9.9 %
		Sector or trade union	276	30.6 %
		Company union	26	2.9 %

Frequencies, Totals, and Proportions by Category.

The table further categorizes the maximum level of union organization participating in the strikes. Sector or trade unions were the most frequently involved (30.6 %), followed by socio-union movements (19.8 %), trade union centers (16.4 %), and confederations (14.2 %). Temporary macro-organizations (6.3 %) and company unions (2.9 %) were less commonly represented. Additionally, the dataset includes information on the names of the calling organizations (e.g., specific trade unions) and the political parties supporting the call for a general strike, though these details are not presented in the table.

The communication variables (Table 3) capture key aspects of dissemination and negotiation efforts during political strikes. Public statements were issued in 53.6 % of events, highlighting their role in shaping narratives and garnering support. Negotiation tables were convened in 25.3 % of cases, reflecting efforts to engage in dialogue and resolve conflicts. Press conferences and central statements were reported in 13.0 % of events, indicating their relatively limited but strategic use in conveying the demands and positions of strike organizers.

**Table 3:** Communications variables.

Variable	Description	Category	Frequency	Proportion
declaraciones	Public statements	1 = Yes	517	53.6 %
		2 = No	448	46.4 %
mesa	Indicates whether a negotiation table was called for.	1 = Yes	244	25.3 %
		2 = No	721	74.7 %
puntoprensa	Press point and central statement	1 = Yes	125	13.0 %
		2 = No	840	87.0 %

Frequencies, Totals, and Proportions by Category

The geographic and economic sector variables (Table 4) provide detailed insights into the scope and sectoral involvement of political strikes. The geographic scale of strikes ranges from localized actions at the commune level (2.0 %) to large-scale national mobilizations (58.7 %), reflecting the varied reach of these events.

**Table 4:** Geographic and economic sector of strike variables.

Variable	Description	Category	Frequency	Proportion
escala	Geographic scale of the strike	Commune	19	2.0 %
		City, Province, Region	253	26.2 %
		Interregional	126	13.1 %
		National	566	58.7 %
rama1	Main economic sector involved	Agriculture, Forestry, and Livestock	107	11.2 %
		Mining	100	10.4 %
		Manufacturing Industry	23	2.4 %
		Electricity, Water, and Sanitation Services	11	1.1 %
		Construction	15	1.6 %
		Trade	8	0.8 %
		Transportation and Communications	140	14.6 %
		Banking and Financial Services	16	1.7 %
		Central, Regional, and Municipal Government	115	12.0 %
		Education	143	14.9 %
		Health	57	5.9 %
		Social and Personal Services	2	0.2 %
		All sectors	86	9.0 %
		Not mentioned	135	14.1 %
tamano5	Estimated size of mobilization ('at least…')	Hundreds (100–999)	102	10.8 %
		Thousands (1000–9999)	405	43.0 %
		Ten thousands (10,000–99,999)	304	32.3 %
		Hundreds of thousands (100,000–999,999)	113	12.0 %
		Millions	18	1.9 %

Frequencies, Totals, and Proportions by Category.

In terms of economic sectors, strikes were most prominent in education (14.9 %), transportation and communications (14.6 %), and government services (12.0 %), highlighting their substantial societal impact. While Table 4 focuses on primary economic sectors, the dataset also captures secondary and tertiary sectors involved, as well as details on the main, second, and third cities associated with the strikes (not included in the table).

The dataset further estimates the size of mobilizations based on news reports, categorizing participation into five ranges: hundreds (100–999; 10.8 %), thousands (1000–9999; 43.0 %), ten thousands (10,000–99,999; 32.3 %), hundreds of thousands (100,000–999,999; 12.0 %), and millions (1.9 %). These classifications offer a comprehensive perspective on the geographic reach, economic focus, and scale of political strikes across the region.

The tactics and targets variables (Table 5) provide detailed insights into the primary objectives and methods associated with political strikes. Among the key targets identified, the most frequently mentioned were the government or president (41.3 %), constitution, laws, or regulations (49.6 %), and public or social policies (21.9 %). These figures highlight the significant focus of political strikes on influencing institutional frameworks and policy decisions. Other targets, such as local authorities (6.9 %), ministers (7.8 %), and political parties (0.5 %), were less commonly addressed.

**Table 5:** Tactics and targets variables.

Variable	Description	Category	Frequency	Proportion
blanco1	Government or President	1 = Yes	399	41.3 %
		2 = No	566	58.7 %
blanco2	Constitution, laws or regulations	1 = Yes	479	49.6 %
		2 = No	486	50.4 %
blanco3	Public or social policy	1 = Yes	211	21.9 %
		2 = No	754	78.1 %
blanco4	Congress or Chambers	1 = Yes	22	2.3 %
		2 = No	943	97.7 %
blanco5	Minister(s)	1 = Yes	75	7.8 %
		2 = No	890	92.2 %
blanco6	Political party(ies)	1 = Yes	5	0.5 %
		2 = No	960	99.5 %
blanco7	Local authorities	1 = Yes	67	6.9 %
		2 = No	898	93.1 %
tactica1	Main mobilization tactic	Marches	290	30.1 %
		Protest or demonstration points	161	16.7 %
		Pickets outside companies	8	0.8 %
		Territorial strike support committees	1	0.1 %
		Historical event commemorations	1	0.1 %
		Artistic-cultural group displays	1	0.1 %
		Commercial boycott campaigns	13	1.3 %
		Road blockages	104	10.8 %
		Occupations of state buildings	11	1.1 %
		Occupations of companies	2	0.2 %
		Occupations of open public spaces	6	0.6 %
		Self-harm actions (or threats of) (hunger strikes, self-immolation)	3	0.3 %
		Private property destruction	1	0.1 %
		Public property destruction	2	0.2 %
		Barricades	5	0.5 %
		Assault on repressive forces	28	2.9 %
		Judicial actions	1	0.1 %
		Ethical shifts	2	0.2 %
		Sit-down or slow-down strikes	28	2.9 %
		Production sabotage	20	2.1 %
		Not mentioned	277	28.7 %

Frequencies, Totals, and Proportions by Category.

The dataset also captures the diverse array of tactics employed during these strikes. A total of 20 distinct tactics where coded, showcasing the variety of strategies used by organizers. The most common tactics were marches (30.1 %), protests or demonstration points (16.7 %), and road blockages (10.8 %), reflecting the widespread use of mass mobilizations and disruptive methods. While Table 5 focuses on primary tactics, the dataset also records secondary and tertiary tactics utilized during the strikes (not reported in the table).

The repression variables (Table 6) provide insights into the presence and nature of repressive measures observed during political strikes. Police presence was recorded in 35.9 % of events, emphasizing the significant role of law enforcement in managing these mobilizations. Associated variables include the occurrence of arrests (18.7 %), injuries to protesters (14.0 %), and fatalities (13.3 %), indicating varying levels of police intervention and its potential consequences.

**Table 6:** Repression variables.

Variable	Description	Category	Frequency	Proportion
arrestos1	Presence of arrested protesters	1 = Yes	180	18.7 %
		2 = No	785	81.3 %
heridos1	Presence of injured protesters	1 = Yes	135	14.0 %
		2 = No	830	86.0 %
muertes1	Presence of deceased protesters	1 = Yes	128	13.3 %
		2 = No	837	86.7 %
policia	Presence of police forces	1 = Yes	346	35.9 %
		2 = No	619	64.1 %
repleg	Legal repression: complaints (or threats of) against leaders	1 = Yes	75	7.8 %
		2 = No	890	92.2 %
repleg2	Legal repression: preventive detention of leaders	1 = Yes	33	3.4 %
		2 = No	932	96.6 %
repleg3	Legal repression: invoking national security laws	1 = Yes	11	1.1 %
		2 = No	954	98.9 %
repleg4	Legal repression: State of Emergency	1 = Yes	49	5.1 %
		2 = No	916	94.9 %

Frequencies, Totals, and Proportions by Category.

In addition to physical repression, the dataset captures instances of legal repression, although such measures were less frequent. Preventive detention of leaders was observed in 3.4 % of cases, while the invocation of national security laws occurred in only 1.1 % of events. Other legal repressive actions, such as declaring a state of emergency (5.1 %) or filing legal complaints or threats against strike leaders (7.8 %), further illustrate the institutional responses to political strikes.

These variables collectively highlight the dual dimensions of repression—physical and legal—employed during political strikes in Latin America and of how states respond to these labor mobilizations.

The demands and consequences variables (Table 7) capture the core objectives and political outcomes associated with political strikes. The dataset codes a total of 47 distinct demands, and for events with multiple demands, it differentiates between primary, secondary, and tertiary demands (only primary demands are reported in Table 7). The most frequent primary demands include those related to remuneration policies (26.9 %), economic policy (14.1 %), and changes to laws or decrees (16.7 %). These demands reflect the diverse issues at the heart of political strikes, ranging from immediate labor-related grievances to broader economic and legal reforms.

**Table 7:** Demands and consequences of political strike.

Variable	Description	Category	Frequency	Proportion
demanda1	Main demand	Not mentioned	9	0.9 %
		Related to remuneration policies (salary, bonuses, unpaid wages, etc.)	260	26.9 %
		Mistreatment	1	0.1 %
		Align conditions between permanent and subcontracted employees	2	0.2 %
		Non-compliance with agreements	22	2.3 %
		Related to the negotiation process	2	0.2 %
		Change in company ownership	2	0.2 %
		Solidarity	1	0.1 %
		Anti-union practices	2	0.2 %
		Support for union leaders	7	0.7 %
		Against union leadership	1	0.1 %
		Working conditions, hygiene, and safety	34	3.5 %
		Greater labor inspection	5	0.5 %
		Intervene in companies	4	0.4 %
		State aid (subsidies, avoiding fines)	27	2.8 %
		Greater governance in the local environment, public security	11	1.1 %
		State facilities to the local community (permits, autonomy, etc.)	8	0.8 %
		Increase or improve social spending (housing, health, roads, etc.)	36	3.7 %
		To influence the design of the project or social policy	3	0.3 %
		Responses to socio-environmental or environmental damages	14	1.5 %
		Against privatization policy	42	4.4 %
		Against labor reform	15	1.6 %
		Minimum wage increase	17	1.8 %
		Nationalization of natural resources	4	0.4 %
		Against or in favor of laws or decrees	161	16.7 %
		Constitutional Assembly	6	0.6 %
		Support for political authorities	3	0.3 %
		Layoffs	35	3.6 %
		Resignation/dismissal of the President	25	2.6 %
		Resignation/dismissal of Minister(s)	6	0.6 %
		Against other political authorities	11	1.1 %
		Against other State bodies	2	0.2 %
		Against repression	8	0.8 %
		Against official State reports (their legitimacy)	2	0.2 %
		Against election procedures or results	12	1.2 %
		Economic policy (employment, trade agreements, goods prices, etc.)	136	14.1 %
		Inflation and/or its management, cost of living	6	0.6 %
		Greater participation in national wealth, better distribution	12	1.2 %
		More resources for the locality or region	1	0.1 %
		Workday structure	1	0.1 %
		Total workday time	1	0.1 %
		Irregularities committed by coworkers	1	0.1 %
		Productive organization	4	0.4 %
		Lack of supplies	2	0.2 %
		Lack of personnel	1	0.1 %
efautoridad	Resignation or dismissal of local authority	1 = Yes	9	0.9 %
		2 = No	956	99.1 %
efgabinete	Cabinet reshuffle	1 = Yes	24	2.5 %
		2 = No	941	97.5 %
efministro1	Resignation or dismissal of ministers	1 = Yes	61	6.3 %
		2 = No	904	93.7 %
efministro2	Impeachment of ministers	1 = Yes	13	1.3 %
		2 = No	952	98.7 %
efpresidente1	Presidential resignation	1 = Yes	8	0.8 %
		2 = No	957	99.2 %
efpresidente2	Impeachment of the president	1 = Yes	8	0.8 %
		2 = No	957	99.2 %

Frequencies, Totals, and Proportions by Category.

Regarding consequences, the dataset identifies seven potential political outcomes resulting from strikes. Among the notable outcomes are cabinet reshuffles (2.5 %) and the resignation of ministers (6.3 %), underscoring the potential impact of strikes on governmental structures. Additionally, the dataset accounts for less common outcomes, such as the closure of parliament or chambers and the resignation or dismissal of local authorities. These outcomes, while rare, highlight the broader political ramifications that strikes can have on institutional stability.

The Social Context Variables (Table 8) capture key external and domestic factors shaping the environment in which political strikes occur. Among external factors, the presence of economic crises was recorded in 22.5 % of events, highlighting the influence of macroeconomic instability on labor mobilization. Political reform processes, noted in 9.2 % of cases, provide insight into institutional changes that may trigger or coincide with strikes. Additionally, socio-environmental crises were rare, appearing in only 0.5 % of events.

**Table 8:** Social context variables.

Variable	Description	Category	Frequency	Proportion
crisisecol	Presence of Socio-environmental problem or crisis	1 = Yes	5	0.5 %
		2 = No	960	99.5 %
crisisecon	Presence of Economic crisis	1 = Yes	217	22.5 %
		2 = No	748	77.5 %
ideogob	Political ideology of the government	Left	176	18.2 %
		Center-left	282	29.2 %
		Center	129	13.4 %
		Center-right	300	31.1 %
		Right	78	8.1 %
protext	Presence of External protests	Wave of protests	284	29.4 %
		Isolated protests	368	38.1 %
		No previous protests	313	32.4 %
reflab	Presence of Labor reform process	1 = Yes	47	4.9 %
		2 = No	918	95.1 %
refpol	Presence of Political reform process	1 = Yes	89	9.2 %
		2 = No	876	90.8 %

Frequencies, Totals, and Proportions by Category.

Domestic political context is also well-represented in the dataset. The political ideology of the government is categorized across a spectrum—left (18.2 %), center-left (29.2 %), center (13.4 %), center-right (31.1 %), and right (8.1 %)—offering a nuanced view of how political orientation may influence or respond to strikes. Labor reform processes were present in 4.9 % of events, reflecting instances where labor-related policy changes intersected with mobilization.

Furthermore, the dataset includes the presence of external protests, distinguishing between waves of protests (29.4 %), isolated protests (38.1 %), and no previous protests (32.4 %). To provide additional political context, the database also records the name of the country’s president at the time of the strike, enriching the dataset with a layer of historical and institutional specificity.

## 4 Experimental Design, Materials and Methods

The dataset was constructed using Protest Event Analysis [6] and quantitative content analysis [7] methodologies, enabling the systematic coding of political events based on newspaper data. The coding process was carried out in four distinct steps.

### 4.1 Event identification stage and data collection

The process of identifying political strikes involved a meticulous approach, leveraging a paid subscription to *LatinNews*, a comprehensive news service covering Latin America and the Caribbean. To capture the full spectrum of events associated with political strikes, a carefully curated list of keywords was utilized. These keywords included general terms related to labor activism as well as more specific terms describing various worker actions. This ensured that the search encompassed a wide range of relevant activities and incidents.

The data collection involved several key steps:

a. **Advanced Search:**
  A preliminary search was conducted using five carefully selected keywords: *strike, stoppage, protest, labor unrest*, and *labor mobilization*. These terms were applied to news articles published between 1990 and 2020. The search aimed to maximize coverage of political strike events by identifying potentially relevant press releases.
b. **Manual Review:**
  Following the initial keyword search, manual reviews were conducted to refine the dataset. This process involved systematically examining reports from the *Latin American Weekly Report* and *LatinNews Daily* for the years flagged by the search. This manual step was essential for verifying the relevance of each event and ensuring the dataset’s comprehensiveness.

The data collection phase, carried out between June and August 2022, yielded 3311 press releases. From this pool, 965 events were identified as political strikes. These strikes were defined as labor mobilizations targeting government policies rather than employers, focusing on influencing public policies or decisions made by political authorities. This distinction is critical in differentiating political strikes from economic strikes.

One of the challenges in coding strikes arises in cases involving public sector employees. While it may appear that all public-sector strikes are inherently political—since they target the government as the representative of the state—this is not always accurate. Some strikes by public employees are directed at the state in its capacity as an employer and are therefore categorized as economic strikes.

For example: a strike by public employees demanding better salary agreements is classified as an economic strike, as the conflict targets the government as an employer. If the conflict requires the government to modify or reorient its public policies to improve working conditions, it is classified as a political strike, as the target is the government as a policymaker.

To ensure the accuracy and reliability of the dataset, all events initially coded as political strikes underwent a secondary review by the principal investigator. This additional verification step was critical for maintaining consistency in distinguishing between economic and political strikes.

### 4.2 Codebook design

The design of the codebook was a critical step in standardizing the coding and analysis of the newspaper archives. The codebook provided clear definitions and explicit rules to ensure consistency and reliability in the coding process. Its development was grounded in pre-existing theoretical frameworks but also incorporated an inductive component, drawing insights from the political strikes identified in the collected news reports. This combination of deductive and inductive approaches allowed for a comprehensive and context-sensitive tool tailored to the shades of political strikes in the region.

The codebook comprises 73 variables, systematically organized into key thematic sections (see the Data Description section). The codebook was the essential tool for transforming unstructured information from newspaper archives into a robust and analyzable dataset.

### 4.3 Coding process

The coding phase, conducted from September 2022 to July 2023, was a meticulous and collaborative effort aimed at transforming qualitative data from news reports into a structured and analyzable dataset. The process involved three trained coders who worked systematically to manage the extensive volume of data, ensuring accuracy and consistency throughout the coding phase.

To streamline the workload, the political strikes were divided by country among the coders. This division allowed each coder to focus on specific national contexts, fostering a deeper understanding of the local dynamics and nuances of the events. The coders underwent rigorous training to familiarize themselves with the definitions, rules, and guidelines outlined in the codebook. This preparation was crucial for ensuring a standardized approach across all coders, minimizing discrepancies in the interpretation and classification of data.All coder adhered to the same methodological rigor applied, ensuring the integrity of the dataset across the entire temporal span.

Regular meetings were held among the coders and the principal investigator to review progress, address ambiguities, and resolve inconsistencies. These collaborative discussions were integral to maintaining the reliability of the data and ensuring that the coding adhered to the theoretical framework and operational definitions established in the codebook.

### 4.4 Validation process

The validation process for the dataset involved two key strategies: intercoder reliability testing and cross-referencing with external databases. This multi-pronged approach ensured the accuracy, consistency, and robustness of the data.

### 4.5 Intercoder reliability and internal validation

To assess and enhance the reliability of the coding process, a resampling procedure was conducted, covering 20 % of local and sectoral strikes. Initial intercoder reliability was measured at 92 %, reflecting a high degree of agreement among coders. Any discrepancies or missing data identified during this validation process were addressed through additional searches in the *LatinNews* archives. Disagreements between coders or datasets were collectively reviewed and resolved through consensus, ensuring consistency in the final dataset.

### 4.6 Cross-Validation with external databases

Although this dataset represents the first systematic database of political strikes in Latin America, external sources were used to validate its comprehensiveness and accuracy. Two major external databases were employed for this purpose:

1. **The Cross-National Time-Series (CNTS) Database** [8]**:**
  The CNTS database includes a variable on general strikes, which records the frequency of strikes involving >1000 workers directed against the state across multiple countries. While this variable provided valuable context, it presented several limitations when compared to the political strike definition adopted in this study:
  ○ The CNTS variable does not exclude public-sector strikes targeting the government in its capacity as an employer, which are classified as economic strikes in our dataset.
  ○ It omits strikes involving fewer than 1000 workers, thereby missing smaller but potentially significant political mobilizations.
  ○ The database focuses solely on strike frequency without capturing internal or external characteristics of the strikes.
    Despite these limitations, the CNTS database served as a useful comparative tool for validating general strike frequencies across countries.
2. **The Martínez Database on Political Events** [9]**:**
  This dataset, which includes variables on presidential scandals, anti-government demonstrations, and general strikes in Latin America, offered additional insights for external validation. While it provided valuable data on anti-government protests and strikes, it was not specifically designed to codify general strikes, limiting its applicability. Nevertheless, it allowed for a country-level comparison of general strike frequencies, contributing to the validation process.

Each strike identified in the CNTS or Martínez databases but absent from our dataset was thoroughly researched and validated through additional sources. Conversely, strikes present in our dataset but missing from the external databases were carefully reviewed to confirm their validity. This bidirectional validation process strengthened the dataset's reliability and ensured that no significant events were overlooked.

By combining intercoder reliability testing with external database cross-referencing, the validation process enhanced the robustness and credibility of this pioneering dataset on political strikes in Latin America.

## Limitations

The dataset on political strikes in Latin America (1990–2020) is a significant contribution to the study of labor mobilizations in the region, yet it is not without limitations. These constraints primarily stem from the methodology and sources used for data collection and coding.

The dataset was constructed using *LatinNews* as the primary source for data collection, which required a labor-intensive manual review of bulletins. While this approach allowed for a detailed analysis of reported events, it also introduced the possibility of missing political strikes that were not explicitly labeled or reported as such in the archive. Events that fell outside the scope of the predefined keywords or were ambiguously described may not have been captured.

As a press-based dataset, the reliance on media coverage may introduce selection bias. News outlets often prioritize larger or more politically significant events, which could lead to the underreporting of smaller-scale political strikes. This limitation is particularly relevant for local political strikes, which may have received limited media attention during the period of study. However, the dataset provides a robust representation of general and sectoral political strikes, which are typically better documented in press reports.

Finally, although the coding process included rigorous training and resampling measures to ensure consistency, the subjective nature of interpreting and categorizing complex events may introduce a degree of coder bias. Initial intercoder reliability was high (93 %), and any discrepancies were addressed through systematic validation and review. Nevertheless, the inherent challenges of coding political events remain a potential source of variability.

## Ethics Statement

The authors have read and followed the ethical requirements for publication in Data in Brief and confirmed that the current work does not involve human subjects, animal experiments, or any data collected from social media platforms.

## CRediT Author Statement

Not Applicable to a single-authored.

## Data Availability

DataversePolitical Strikes in Latin America (1990–2020) (Original data).
